# Longitudinal automated assessments of myocardial strain from cine MR in cancer patients receiving cardiotoxic chemotherapy

**DOI:** 10.1186/1532-429X-18-S1-O81

**Published:** 2016-01-27

**Authors:** Marie-Pierre Jolly, Jennifer H Jordan, Craig A Hamilton, Gregory Hundley

**Affiliations:** 1 Medical Imaging Technologies, Siemens Healthcare, Princeton, NJ USA; 2 Cardiovascular Medicine, Wake Forest School of Medicine, Winston-Salem, NC USA; 3 Biomedical Engineering, Wake Forest School of Medicine, Winston-Salem, NC USA; 4 Radiology, Wake Forest School of Medicine, Winston-Salem, NC USA

## Background

The use of chemotherapy for the treatment of cancer is associated with cardiovascular injuries and is most commonly monitored with assessments of left ventricular ejection fraction (EF) [1]. Often, EF changes are detected quite late; myocardial strain may identify early, subclinical changes in myocardial injury prior to a decline in EF. Recently, it has been possible to measure myocardial strain directly from cine images without the need for tagged images. We performed a longitudinal study to determine (1) the feasibility of assessing myocardial strain from cine images with automated analysis and (2) if subclinical changes in myocardial contractility can be observed with this new technique in patients receiving cardiotoxic chemotherapies.

## Methods

Segmented SSFP cine imaging was performed on a 1.5T Magnetom Avanto (Siemens Healthcare GmbH, Erlangen) in a total of 57 patients (70% treated with anthracyclines). A baseline scan was acquired after cancer diagnosis and prior to chemotherapy initiation. A second scan was acquired after three months of chemotherapy treatment. Myocardial contours were automatically generated on the short axis slices with a prototype (Trufi Strain, Siemens Healthcare GmbH, Erlangen) based on an previously described automatic algorithm using deformable registration [2, 3]. Peak circumferential Lagrangian strain at the midline inside the myocardium in the mid-ventricular slice is then automatically calculated from the tracked myocardial contours. Left ventricular volume and EF were quantified using the same contours. CMR values from the baseline visit and the 3-month visit were then compared using paired t-tests in SAS (SAS Institute Inc, Cary, NC).

## Results

**Figure 1:**
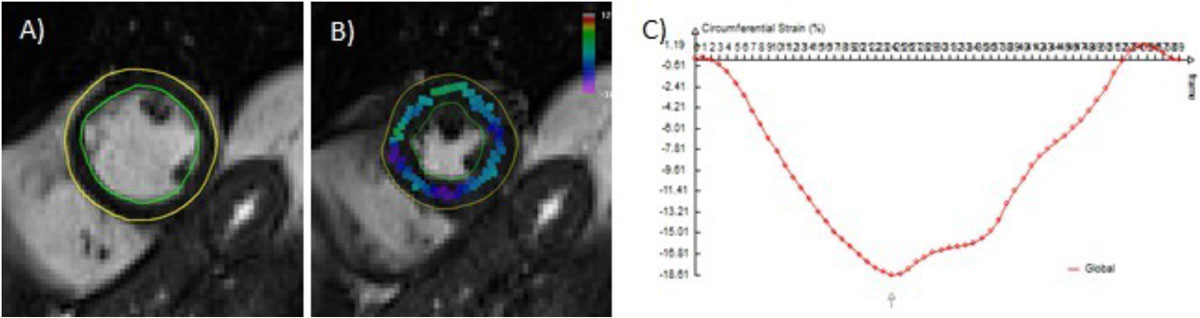
**Automatic calculation of strain from cine images**: A) Automatically generated myocardial contours; B) Mid ventricular mid myocardial peak circumferential strain; C) Circumferential strain curve over the cardiac cycle.

**Figure 2:**
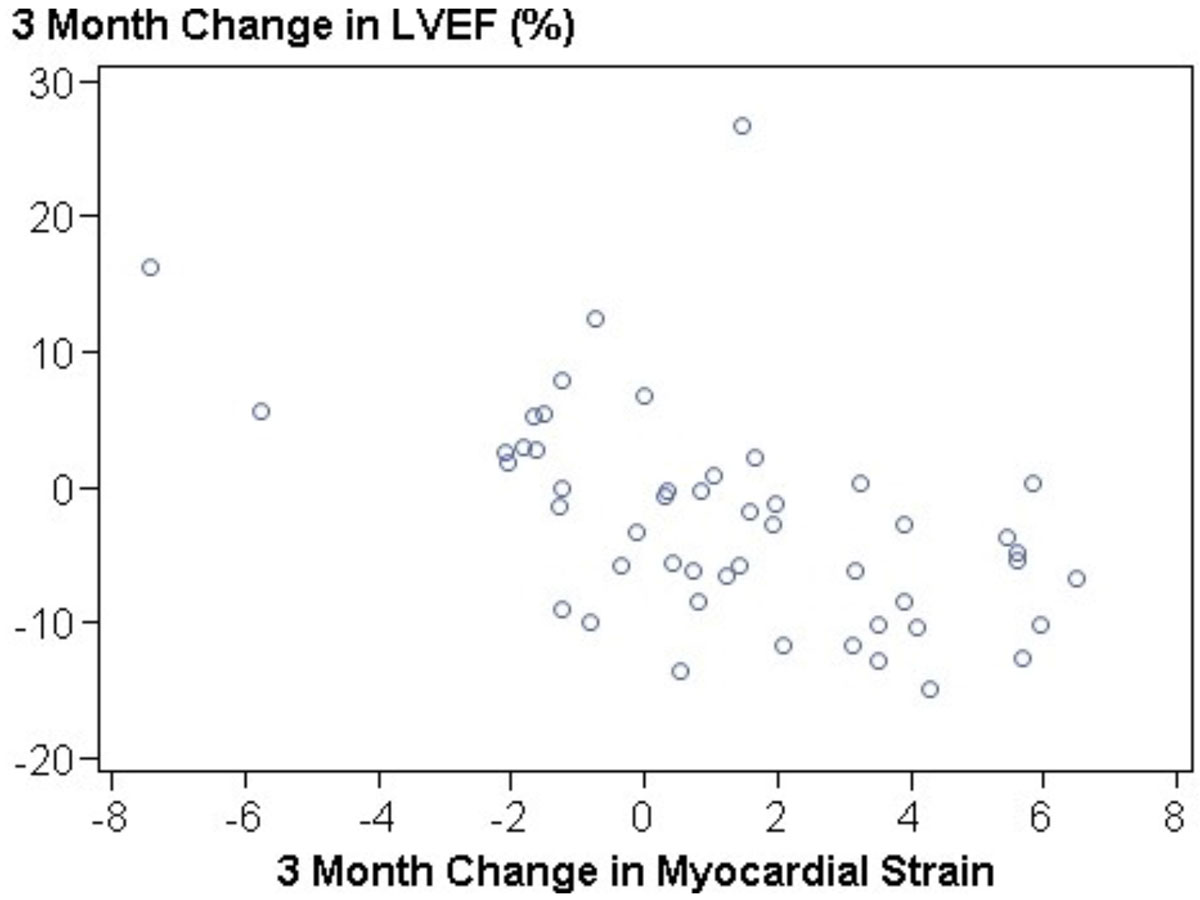
**Longitudinal increases in myocardial strain, showing less contractility, are inversely correlated with subclinical reductions in left ventricular ejection fraction (LVEF) using assessments from automated contouring and analysis of cine SSFP imaging in a cohort of cancer patients receiving potentially cardiotoxic chemotherapy (p = 0.0457)**.

Eight out of the 114 automatic contouring (7%) were unsuccessful and these cases were not considered for further evaluation; a total of 49 paired assessments were thus included in analyses (Figure 1). Myocardial strain became less negative (changing from -18.7 ± 2.6 to -17.4 ± 2.9 [p = 0.0054]) following chemotherapy. Left ventricular EF decreased from 62.1 ± 8.2% to 59.7 ± 7.7% during this same time period (p = 0.0457). As shown in Figure 2, changes in myocardial strain were inversely associated with subclinical decreases in left ventricular EF (r = -0.53, p < 0.0001).

## Conclusions

These results demonstrate that automated analysis of myocardial strain with deformable registration is feasible in clinical applications. Furthermore, we observed a decrease in circumferential strain after only 3-months of cardiotoxic chemotherapy treatment in the setting of preserved and mildly reduced EF.
